# Assessment of the Toxicity of Biocompatible Materials Supporting Bone Regeneration: Impact of the Type of Assay and Used Controls

**DOI:** 10.3390/toxics10010020

**Published:** 2022-01-06

**Authors:** Milena Chraniuk, Mirosława Panasiuk, Lilit Hovhannisyan, Sabina Żołędowska, Dawid Nidzworski, Lidia Ciołek, Anna Woźniak, Agnieszka Kubiś, Natalia Karska, Zbigniew Jaegermann, Sylwia Rodziewicz-Motowidło, Monika Biernat, Beata Gromadzka

**Affiliations:** 1 Department of In Vitro Studies, Institute of Biotechnology and Molecular Medicine, Kampinoska 25, 80-180 Gdańsk, Poland; m.panasiuk@ibmm.pl (M.P.); l.hovhannisyan@ibmm.pl (L.H.); s.zoledowska@ibmm.pl (S.Ż.); d.nidzworski@ibmm.pl (D.N.); 2 Biomaterials Research Group, Ceramic and Concrete Division in Warsaw, Łukasiewicz Research Network-Institute of Ceramics and Building Materials, Cementowa 8, 31-983 Kraków, Poland; lidia.ciolek@icimb.lukasiewicz.gov.pl (L.C.); anna.wozniak@icimb.lukasiewicz.gov.pl (A.W.); zbigniew.jaegermann@icimb.lukasiewicz.gov.pl (Z.J.); monika.biernat@icimb.lukasiewicz.gov.pl (M.B.); 3 Department of Biomedical Chemistry, Faculty of Chemistry, University of Gdańsk, Wita Stwosza 63, 80-308 Gdańsk, Poland; agnieszka.szczepanik@phdstud.ug.edu.pl (A.K.); natalia.karska@ug.edu.pl (N.K.); s.rodziewicz-motowidlo@ug.edu.pl (S.R.-M.)

**Keywords:** toxicity of biomaterials, multicomponent biocomposites, experimental controls, GLP for toxicity in vitro studies

## Abstract

Assessing the toxicity of new biomaterials dedicated to bone regeneration can be difficult. Many reports focus only on a single toxicity parameter, which may be insufficient for a detailed evaluation of the new material. Moreover, published data frequently do not include control cells exposed to the environment without composite or its extract. Here we present the results of two assays used in the toxicological assessment of materials’ extracts (the integrity of the cellular membrane and the mitochondrial activity/proliferation), and the influence of different types of controls used on the obtained results. Results obtained in the cellular membrane integrity assay showed a lack of toxic effects of all tested extracts, and no statistical differences between them were present. Control cells, cells incubated with chitosan extract or chitosan-bioglass extract were used as a reference in proliferation calculations to highlight the impact of controls used on the result of the experiment. The use of different baseline controls caused variability between obtained proliferation results, and influenced the outcome of statistical analysis. Our findings confirm the thesis that the type of control used in an experiment can change the final results, and it may affect the toxicological assessment of biomaterial.

## 1. Introduction

The progressive development of regenerative medicine contributed to a significant acceleration of research related to the search and optimization of materials supporting tissue reconstruction. Therefore, increased number of studies on bone regeneration and materials supporting this process are being published [1,2]. The improvement of biocompatible materials is often associated with the addition of enriching substances, such as hydroxyapatite-based ceramics, bioglasses [1], trace element oxides [3], and compounds stimulating proliferation or with antibacterial [4] or anti-inflammatory properties [5,6]. One of the more widely studied solutions are composites composed of chitosan and bioglass [7,8] that are characterized by high biocompatibility [9] or antimicrobial properties [10,11].

Most data on the evaluation of the cytotoxicity of biomaterials supporting bone regeneration composed of chitosan and bioglass found in published reports lack important features. Many reports have not considered the use of the non-cancerous in vitro model [7,8] of human osteoblast lines [12,13]. In addition, published experimental papers most often have not described more than one toxicity parameter [13,14,15], or only qualitative determinations were performed [8].

In this report, we present the results of quantitative toxicity tests based on various molecular mechanisms performed for chitosan-based biocomposites enriched with bioglass, or bioglass and functional peptides. All presented experiments were conducted on healthy human hFOB 1.19 osteoblasts as an in vitro cell model. Our results prove that the type of assay is of key importance for the correct toxicological assessment of new materials. Also, the type of experimental control is relevant for the analysis of obtained results.

## 2. Materials and Methods

### 2.1. Peptide Synthesis and Purification

All of the peptides were synthesized using the standard solid-phase synthesis technique (CEM Corporation, Matthews, NC, USA) on Rink Amide ProTide Resin (capacity 0.21 mmol/g, CEM Corporation, Matthews, NC, USA) according to the Fmoc-chemistry strategy. The peptides were cleaved from the resin, and purified according to the procedure described by Karska and colleagues [16]. The identity of peptides was confirmed by matrix-assisted laser desorption ionization-time of flight mass spectrometry (MALDI-TOF MS, Bruker Daltonics, Bremen, Germany), and electrospray ionization mass spectrometry (ESI-MS, Shimadzu, Kyoto, Japan). Amino acid derivatives and reagents for the synthesis were purchased from Lipopharm (Gdańsk, Poland), whereas the reagents for purification were purchased from Merck (Darmstadt, Germany).

### 2.2. Composites and Bioglasses Preparation

Porous composites developed as materials for bone tissue regeneration were used in this research. Composites were made by the thermal phase separation method based on a natural polysaccharide—chitosan. The commercial product Chitoceuticals from Heppe Medical Chitosan (Halle, Germany) was used. Bioglass based on the sol-gel method was used as a filler for the developed composites. The weight ratio of bioglass and chitosan in the composites was 1:1. The bioglass was prepared using the silica oxide—phosphorus pentoxide—calcium oxide (SiO_2_-P_2_O_5_-CaO) system. The chemical composition of the bioglass (B) includes: 70% wt. SiO_2_, 5% wt. P_2_O_5_, and 23% wt. CaO and 2% wt. SrO. Functional peptides (pro-regenerative: p1, p2, p4, antibacterial: p3) were incorporated into composites by covalent bond or by surface adsorption through dipping composites in peptides solutions and freeze-drying. The percentage of peptides in the composites was: p1-1—2.1765%, p1-2—0.0560%, p-2—0.1750%, p-3—0.0077%, p4-1—0.1594%, p4-2—0.2906%. All chitosan-based biomaterials used in this study were prepared in standardized form—discs of a diameter of ~14.5 mm, and a thickness of 1 mm. Dried discs were sterilized by gamma radiation, and were not weighted before the extraction to maintain sterility of the materials. To obtain the extract, each tested sample was prepared from one disc.

### 2.3. Cell Culture

Human fetal osteoblasts (hFOB 1.19; ATCC CRL-11372, LGC Standards, Kiełpin, Poland) were cultured in a 1:1 mixture of Ham’s F12 Medium and Dulbecco’s Modified Eagle’s Medium (DMEM) with 2.5 mM L-Glutamine (without phenol red) supplemented with 10 µg/mL of gentamicin, 0.25 µg/mL amphotericin B, and Fetal Bovine Serum (FBS) at a final concentration of 10%. The cells were cultured at 34 °C and 5% CO_2_ in a CB240 incubator (Binder, Tuttlingen, Germany). The above-mentioned cell culture medium was used in further experiments. All reagents were purchased from Thermo Fisher Scientific (Waltham, MA, USA). hFOB 1.19 cells were chosen for this study as a cell model due to their biological similarity to primary osteoblasts. The animal cell lines or human cancer cells were excluded from this research due to differences between the biology of animal cells, cancer cells, and the healthy human cells, such as hFOB 1.19.

### 2.4. Extract Preparation

For the extraction of composites, the composites were immersed in 1.5 mL culture medium in a 24-well plate (Sarstedt, Nümbrecht, Germany) at 34 °C and 5% CO_2_. After 24 h incubation, the extracts were collected. On the previous day, hFOB 1.19 cells were seeded at a density of 5 × 10^4^/cm in 24-well plates. After 24 h incubation, the medium was replaced with 1 mL of composite extract or fresh medium, and the plates were incubated for a further 48 h at 34 °C and 5% CO_2_. Each plate contained triplicates of untreated cells, and cells treated with chitosan (CH), chitosan-bioglass (CHB), chitosan-bioglass-peptide (CHBp) extracts, positive control, and blank wells. After the incubation, cell viability and cytotoxicity were measured. The experiment was repeated twice.

### 2.5. Cytotoxicity—Lactate Dehydrogenase (LDH) Activity Assay

The test was performed using a Cytotoxicity Detection Kit^PLUS^ (LDH) (Roche Applied Science, Penzberg, Germany) according to the supplier’s protocol. Briefly, the cells in the 24-well plate were incubated with the extract or fresh medium for 48 h. Collected samples were transferred to 96–well plates (Sarstedt, Nümbrecht, Germany), and mixed with the dye and catalyst solution. After incubation in the dark, the optical density of samples at 490 nm and 690 nm was measured using a plate reader Epoch (BioTek Instruments, Winooski, VT, USA). As a positive control, the cells treated with the Triton—X100 solution included in the kit were used. Untreated cells and blank medium were included into each assay. Values of background absorbance and blank absorbance were subtracted from all the absorbance values.

The percentage of cytotoxicity was calculated by the equation below:

Cytotoxicity [%] = (Sample absorbance − Control absorbance)/(Positive control absorbance − Control absorbance) × 100%

All controls in calculations were means of triplicates.

### 2.6. Proliferation—WST-1 Mitochondrial Activity Assay

The proliferation of hFOB 1.19 cells was evaluated using a WST-1 assay kit (Abcam, Cambridge, UK) according to the manufacturer’s protocol. Briefly, the cells in a 24-well plate were incubated with the extract or fresh medium for 48 h, and then treated with 40 µL of WST-1 reagent, followed by 2 h incubation at 34 °C and 5% CO_2_ in a CB240 incubator (Binder, Tuttlingen, Germany)

The media were collected from each well, and transferred to 96-well flat bottom plate (Sarstedt, Nümbrecht, Germany). The optical density at 450 nm and 620 nm was measured using a plate reader, Epoch (BioTek Instruments, Winooski, VT, USA). The untreated cells and blank were included into each assay. The values of background and blank absorbance were subtracted from all the absorbance values. The percentage of proliferation was calculated by the equation below:

Proliferation [%] = (Sample absorbance/Control absorbance) × 100%

The control absorbance was calculated as a mean of triplicate.

### 2.7. Statistical Analysis

Collected data were analyzed and visualized using GraphPad Prism (GraphPad Software, San Diego, CA, USA). Statistical analysis was caried out using a Kruskal–Wallis test (*p* = 0.05). In the next step, to control the false discovery rate, a Benjamini, Krieger, and Yekutieli multiple comparison test (*p* = 0.05) was conducted. Analyses were performed for medians of data obtained in two technical repeats of the experiment. Input datasets for each type of sample were compared between repeats before the main statistical analysis. Comparison methods included a Kruskal–Wallis test (*p* = 0.05), followed by a Benjamini, Krieger, and Yekutieli multiple comparison test (*p* = 0.05) or a Mann–Whitney test (*p* = 0.05). Only one statistically significant difference was found, and it was detected in CHB datasets obtained in the WST-1 test (*p* = 0.0170). We decided to use the full dataset for CHB in the main statistical analysis because the compared data groups cannot be excluded as the data describing other population due to the small number of CHB composites in the experiment. All data are presented in this work as means along with standard deviations.

## 3. Results

**Figure 1:**
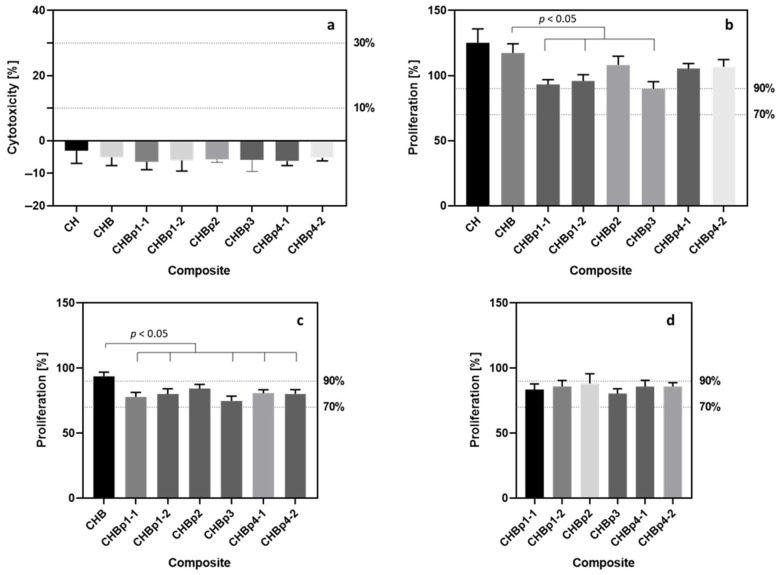
Cytotoxicity (**a**) and proliferation of hFOB 1.19 cells calculated with the use of different controls’ absorbance: (**b**) nontreated control cells, (**c**) cells growing in CH extract, (**d**) cells growing in CHB extract. Data shown include results from two technical repeats of the experiment. Each repeat contained triplicates of untreated cells and cells treated with CH, CHB, CHBp extracts. CHBp extracts were numbered due to the presence of different peptides and their variable concentrations in the composites: p1-1—2.1765%, p1-2—0.0560%, p-2—0.1750%, p-3—0.0077%, p4-1—0.1594%, p4-2—0.2906%. The most important statistically significant differences are marked as *p* < 0.05. Full results of statistical analysis can be found in Supplementary Material (Tables S1–S3).

Healthy osteoblasts—hFOB 1.19 cells—were used to assess the cytotoxic effect of biocomposites using the indirect method (according to ISO 10993-5: 2009 Part 5). Briefly, the cells were incubated with extracts prepared from the tested material: (a) CH—base material: chitosan, (b) CHB—two-component: chitosan-bioglass, and (c) CHBp—three-component: chitosan-bioglass-peptide solid composites. Cell cytotoxicity and cell proliferation were then tested by LDH and WST-1 assays. Due to the fact that hFOB 1.19 cells divide every 36 h, a longer incubation time (48 h) with samples was chosen to allow cells to undergo cell division. Extracts from all tested composites did not induce damage to the cell membrane (examined with the LDH assay); obtained results were normalized to the untreated control cells used as a reference (Figure 1a). Moreover, no statistically significant differences were found between the effects of extracts from the individual materials. The level of mitochondrial activity calculated with the WST-1 assay showed more diverse results between the tested materials than the results obtained in the LDH test. The highest value of mitochondrial activity, and thus the highest cell proliferation, was observed among cells incubated with extracts from CH and CHB composites (Figure 1b). Proliferation values reached the following values: 125.20 ± 10.67% and 117.30 ± 7.12% as compared to the control cells that were not incubated with the extracts (proliferation was arbitrarily set as 100%). Moreover, there were statistically significant differences (*p* < 0.05) in the effect of CH extracts and extracts from all CHBp composites. Statistically significant differences were also detected between the proliferation induced by the extract from the CHB composite and extracts from three-component materials enriched with p1and p3 peptides. None of the extracts caused a decrease in proliferation below 70%, which is the threshold value below which the material is considered toxic according to ISO 10993-5: 2009. Only the extract from the composite enriched with the p3 peptide did not reach a more stringent 90% threshold, and reached the value of 89.81 ± 5.53% compared to the control cells.

For both CH and CHB composites, no cytotoxic effect and increase in cell proliferation compared to the untreated control cells were observed. Due to that, for further analysis, it may be beneficial for the rest of the results to be calculated in relation to those base composites as a “starting value” for all the additional substances added. Using those base composites as a reference can make a clearer picture of the beneficial or negative effect of all enriching peptides in the three-component composites.

When the proliferation values were recalculated in relation to the cells incubated with the CH extract (as a base material of all tested composites), a significant change in the results was observed (Figure 1c). In this case, a cell proliferation exceeding 90% was observed only for the extract from the two-component CHB composite. In addition, extracts from three-component materials enriched with p3 and p1-1 caused a reduction in cell proliferation to the following values: 74.89 ± 3.63% and 77.79 ± 3.58% (when the cell proliferation after incubation with CHBp3 and CHBp1-1 extracts calculated in relation to nontreated cells were as follows: 83.81 ± 5.53% and 93.20 ± 3.67%). Again, statistically significant differences (*p* < 0.05) were found between the extracts from the CHB composite and extracts from materials enriched with p1 and p3 peptides. Also, additional statistically significant differences were detected that were not observed for the calculations based on the control cells incubated in a normal culture medium. The differences occurred between the effect of the extract of the two-component composite and the effect of extracts from composites enriched with p4 (*p* = 0.0077).

Statistical analysis of the results of proliferation calculated against cells grown in the pure culture medium showed the existence of statistically significant differences between pairs of extracts from composites enriched with p1-1 and p2, p3 and p2, as well as p3 and p4-2. After calculating the proliferation against the cells incubated with the CH extract, statistically significant differences were detected only for the pair of p3 and p2 extracts. The proliferation results determined against the cells incubated with the two-component composite did not differ from each other for all peptide-enriched composites (Figure 1d).

## 4. Discussion

As more and more complex, multi-component biocompatible materials are being developed, due to various mechanisms of action that are associated with the properties of added ingredients, their cytotoxic effect must be properly evaluated. We suggest, especially for new materials, to use quantitative toxicity tests based on various molecular mechanisms, and to present the results in a standardized form. All results obtained in various tests assessing the survival of the cell model or proliferation should be calculated as a percentage in relation to the used controls instead of the raw data. By showing only absorbance or fluorescence values [15,17,18], the results cannot be fully related to other data found in the literature. To fully assess the effects caused by a material or extract, it is necessary to use a control, e.g., cells that have not been in contact with the composite or extract [13,19]; also, the physiological state of the used cell model should be taken into consideration [20], which, in many published studies for various bio composites, is not counted [7,21,22]. For this purpose, the use of tests with a positive control (e.g., cells treated with Triton X-100 solution for an LDH assay) that will allow the assessment of the condition of the cells during the experiments, is recommended. The omission of the control that hasn’t been in contact with the material or the extract [14,23], and the lack of an assessment of the in vitro model’s condition mean that the properties of tested materials cannot be clearly determined. This approach will provide more transparent information, and will accelerate the refinement of tissue engineering solutions.

Many studies use the direct method in which the material is in immediate contact with the cell model to assess the cytotoxic effect of the material [15,17] (direct method according to ISO 10993-5: 2009 Part 5). In some cases, however, assessing the toxicity of materials used in tissue regeneration can be difficult. This may be the case in tests performed on the material characterized by, e.g., high viscosity, absorbency, or materials of low density that are floating in the cell medium. Elimination of these issues is possible by using the indirect method based on extracts prepared from tested materials. The indirect method can be successfully used to assess the toxicity or pro-regenerative properties of substances released into the environment from the tested biomaterials, [12], but not to the molecular structure of the material.

In this study, no clear differences between the tested extracts from chitosan-based composites were observed in the LDH test examining the damage to the cell membrane. Additionally, a slight cytoprotective effect was observed that could be caused by the presence of chitosan particles in the extracts [24,25]. In a different study where chitosan composites were tested using mouse Sertoli cells (TM4), bone marrow mesenchymal stromal cells (HS-5), and human embryonic kidney 293 cells (HEK293) seeded directly on the material, a 10% cytotoxic effect was observed for each cell line [20]. The differences in these results could be caused by the use of other methods of contact with the material (direct and indirect method according to ISO 10993-5: 2009), as well as the use of different cell lines.

The lack of differences in the results in one assay does not necessarily translate into similar phenomena in tests based on different molecular mechanisms, for example, mitochondrial activity as presented in this article. For both assays used (WST-1, LDH), however, no toxic effects induced by the extracts were noted (proliferation > 70%, cytotoxicity < 30% according to ISO 10993-5: 2009 Part 5). Proliferation exceeding 100% occurred after cells were exposed to chitosan, and chitosan enriched with bioglass extracts. A similar effect to that was observed by Ge et al. for extracts from chitosan enriched with bioglass [12]. Many previous reports, as well as the data shown in this study, indicate that chitosan- and bioglass-based biocomposites present great potential in bone regeneration. Moreover, the addition of the proregenerative peptides can improve the effectiveness of bone healing [26]. However, for many antimicrobial peptides, their biological activity is not targeting only bacterial cells, but may also negatively impact eukaryotic cells [27]. In our study, this phenomenon was also observed for the extract from biocomposites functionalized with the p3 antibacterial peptide.

## 5. Conclusions

The preparation of results in a standardized form requires the calculation of toxicity parameters relative to controls. As mentioned earlier, published research papers do not always refer to the control cells, e.g., the cells that were not in contact with the material or extract. In such cases, the values of proliferation, for example, are determined relative to the material with the least complex composition [28,29]. The data presented in this article indicate that this approach may lead to different conclusions depending on the controls. Also omitted is the impact of the base material in comparison to control cells, which is crucial, especially when assessing the pro-regenerative performance of a new material.

A broader analysis of the toxicity of all new biomaterials should compare results obtained by both indirect and direct methods. Using the direct method, in which cells are seeded directly on the material, the molecular structure of the biomaterial can be evaluated. This will make it possible to evaluate the effectiveness of the new composites under conditions more similar to those in vivo. Also, assays based on different molecular mechanisms in different cell compartments (e.g., the cytoplasm, mitochondria, and nucleus) should be considered. The assays chosen for this research measure the final outcome of complex molecular changes happening in the cells due to the contact with a biomaterial or its extract. To further asses the cytotoxic effects observed in those assays, the quantification of different toxicity markers is necessary.

## Data Availability

The data presented in this study are available on request from the corresponding author. The data are not publicly available due to legal restrictions.

## Supplementary Materials

The following are available online at https://www.mdpi.com/article/10.3390/toxics10010020/s1, Table S1: Results of the statistical analysis of cell proliferation of cells incubated with composites extracts. Control cells cultured in cell media were used as a baseline for the analysis, Table S2: Results of the statistical analysis of cell proliferation of cells incubated with composites extracts. Cells incubated with the chitosan extract were used as a baseline for the analysis, Table S3: Results of the statistical analysis of cell proliferation of cells incubated with composites extracts. Cells incubated with the chitosan-bioglass extract were used as a baseline for the analysis.
