# Supplementary material for: Assessment of the Toxicity of Biocompatible Materials Supporting Bone Regeneration: Impact of the Type of Assay and Used Controls
Source: Toxics. 2022 Jan 6;10(1):20. doi: 10.3390/toxics10010020 (PMC8778995; doi:10.3390/toxics10010020)
Supplement: Supplementary file 1 [file toxics-10-00020-s001.zip › toxics-1503084-SI.pdf]

toxics

MDPI

# Supplementary Materials: Assessment of the Toxicity of Biocompatible Materials Supporting Bone Regeneration: Impact of the Type of Assay and Used Controls

**Milena Chraniuk, Mirosława Panasiuk, Lilit Hovhannisyan, Sabina Żołędowska, Dawid Nidzworski, Lidia Ciołek, Anna Woźniak, Agnieszka Kubiś, Natalia Karska, Zbigniew Jaegermann, Sylwia Rodziewicz-Motowidło, Monika Biernat and Beata Gromadzka**

**Table S1.** Results of the statistical analysis of cell proliferation of cells incubated with composites extracts. Control cells cultured in cell media were used as a baseline for the analysis.

| Comparison | Difference statistically significant | *p* value |
|---|---|---|
| CH vs. CHB | No | 0.2906 |
| CH vs. CHBp1-1 | Yes | < 0.0001 |
| CH vs. CHBp1-2 | Yes | 0.0001 |
| CH vs. CHBp3 | Yes | < 0.0001 |
| CH vs. CHBp2 | Yes | 0.0457 |
| CH vs. CHBp4-1 | Yes | 0.0197 |
| CH vs. CHBp4-2 | Yes | 0.0331 |
| CHB vs. CHBp1-1 | Yes | 0.0003 |
| CHB vs. CHBp1-2 | Yes | 0.0021 |
| CHB vs. CHBp3 | Yes | 0.0001 |
| CHB vs. CHBp2 | No | 0.1631 |
| CHB vs. CHBp4-1 | No | 0.0782 |
| CHB vs. CHBp4-2 | No | 0.1161 |
| CHBp1-1 vs. CHBp1-2 | No | 0.5251 |
| CHBp1-1 vs. CHBp3 | No | 0.6005 |
| CHBp1-1 vs. CHBp2 | Yes | 0.0457 |
| CHBp1-1 vs. CHBp4-1 | No | 0.0893 |
| CHBp1-1 vs. CHBp4-2 | No | 0.0623 |
| CHBp1-2 vs. CHBp3 | No | 0.4229 |
| CHBp1-2 vs. CHBp2 | No | 0.0936 |
| CHBp1-2 vs. CHBp4-1 | No | 0.1767 |
| CHBp1-2 vs. CHBp4-2 | No | 0.1262 |
| CHBp3 vs. CHBp2 | Yes | 0.0331 |
| CHBp3 vs. CHBp4-1 | No | 0.0623 |
| CHBp3 vs. CHBp4-2 | Yes | 0.0457 |
| CHBp2 vs. CHBp4-1 | No | 0.5326 |
| CHBp2 vs. CHBp4-2 | No | 0.6005 |
| CHBp4-1 vs. CHBp4-2 | No | 0.6005 |

**Table S2.** Results of the statistical analysis of cell proliferation of cells incubated with composites extracts. Cells incubated with the chitosan extract were used as a baseline for the analysis.

| Comparison | Difference statistically significant | p value |
|---|---|---|
| CHB vs. CHBp1-1 | Yes | 0.0004 |
| CHB vs. CHBp1-2 | Yes | 0.0065 |
| CHB vs. CHBp3 | Yes | < 0.0001 |
| CHB vs. CHBp2 | No | 0.1710 |
| CHB vs. CHBp4-1 | Yes | 0.0077 |
| CHB vs. CHBp4-2 | Yes | 0.0077 |
| CHBp1-1 vs. CHBp1-2 | No | 0.4385 |
| CHBp1-1 vs. CHBp3 | No | 0.4319 |
| CHBp1-1 vs. CHBp2 | No | 0.1357 |
| CHBp1-1 vs. CHBp4-1 | No | 0.3968 |
| CHBp1-1 vs. CHBp4-2 | No | 0.4385 |
| CHBp1-2 vs. CHBp3 | No | 0.2170 |
| CHBp1-2 vs. CHBp2 | No | 0.3008 |
| CHBp1-2 vs. CHBp4-1 | No | 0.6823 |
| CHBp1-2 vs. CHBp4-2 | No | 0.7475 |
| CHBp3 vs. CHBp2 | Yes | 0.0233 |
| CHBp3 vs. CHBp4-1 | No | 0.1710 |
| CHBp3 vs. CHBp4-2 | No | 0.2266 |
| CHBp2 vs. CHBp4-1 | No | 0.3369 |
| CHBp2 vs. CHBp4-2 | No | 0.3008 |
| CHBp4-1 vs. CHBp4-2 | No | 0.6823 |

**Table S3.** Results of the statistical analysis of cell proliferation of cells incubated with composites extracts. Cells incubated with the chitosan-bioglass extract were used as a baseline for the analysis.

| Comparison | Difference statistically significant | p value |
|---|---|---|
| CHBp1-1 vs. CHBp1-2 | No | 0.7931 |
| CHBp1-1 vs. CHBp3 | No | 0.7136 |
| CHBp1-1 vs. CHBp2 | No | 0.7136 |
| CHBp1-1 vs. CHBp4-1 | No | 0.9337 |
| CHBp1-1 vs. CHBp4-2 | No | 0.8645 |
| CHBp1-2 vs. CHBp3 | No | 0.2724 |
| CHBp1-2 vs. CHBp2 | No | 0.9557 |
| CHBp1-2 vs. CHBp4-1 | No | 0.9337 |
| CHBp1-2 vs. CHBp4-2 | No | 0.9557 |
| CHBp3 vs. CHBp2 | No | 0.2724 |
| CHBp3 vs. CHBp4-1 | No | 0.4025 |
| CHBp3 vs. CHBp4-2 | No | 0.3001 |
| CHBp2 vs. CHBp4-1 | No | 0.9047 |
| CHBp2 vs. CHBp4-2 | No | 0.9499 |
| CHBp4-1 vs. CHBp4-2 | No | 0.9499 |
